# Risk Factors for Obesity in Five-Year-Old Children: Based on Korean National Health Insurance Service (NHIS) Data

**DOI:** 10.3390/children9030314

**Published:** 2022-02-25

**Authors:** Mi Jin Choi, Hyunju Kang, Jimi Choi

**Affiliations:** 1 Department of Nursing, Chodang University, Muan-gun 58530, Korea; mj061079@gmail.com; 2 College of Nursing, Kangwon National University, Chuncheon-si 24341, Korea; 3 Division of Endocrinology and Metabolism, Department of Internal Medicine, Korea University College of Medicine, Seoul 02841, Korea; jjimchoi@gmail.com

**Keywords:** pediatric obesity, risk factors, health risk behaviors

## Abstract

This study aimed to identify the risk factors for obesity in five-year-old children using data from the database of the Korean National Health Insurance Service. We identified 26,047 children who underwent the sixth screening (at age 5) from the 2017 National Health Screening Program for Infant and Children and for whom data from the fourth screening (at age 3) database and the mothers’ health screening and eligibility database were available. To identify the risk factors of obesity, odds ratios and 95% confidence intervals were calculated by a hierarchical multiple logistic regression. Female sex, a birth weight of over 4 kg, the “caution/refer” remark during developmental screening at ages three and five, maternal obesity, and a middle-level income were risk factors for obesity in the subjects. Good appetite, high consumption of milk, heavy intake of sweet food at age three, speedy eating, irregular meals and snack times, large single-meal quantities, heavy intake of oily and salty food, and not performing physical exercise at age five were also considered significant risk factors. For early intervention efforts to prevent childhood obesity, modifiable behavioral factors and other obesity risk factors identified in this study could be used to target high-risk children and dietary behaviors.

## 1. Introduction

Childhood obesity is a global health challenge [1]. It leads to obesity in adulthood [2,3], subsequently becoming a risk factor for several health conditions, including metabolic and cardiovascular diseases [4,5]. In South Korea, the prevalence rate of obesity in children aged 2 to 18 years was 9.8% in 2017; that of early childhood obesity among children aged 54–60 months and 66–71 months in 2016 was 6.57% and 7.68%, respectively [6,7]. According to a World Health Organization estimate in 2020, 38.9 million (5.7%) children aged 0–5 were overweight/obese [1]. In 2014, the Commission on Ending Childhood Obesity, aimed at children under five years of age, was established, emphasizing the importance of early childhood obesity management [8]. There are two reasons that render tackling obesity in early childhood important. First, obesity has an early onset, occurring even before school age [9]. Rapid early growth becomes a risk factor for subsequent childhood obesity [10]. In a previous study, the early body mass index (BMI) trajectory confirmed an obesity risk of over 50% in children 5–8 years of age who showed a rapid increase in BMI around the age of 3.5 [11]. Second, early childhood is an important period in which behavioral patterns related to diet and activity are shaped. Children’s dietary habits are established around the age of three [12,13], and early childhood is also a time when a healthy lifestyle that lowers the risk of obesity can be established through parents’ influence and the creation of a favorable environment [14,15]. Since obesity is difficult to reverse, prevention is important [16], with early childhood being particularly important in this regard; this is because an early intervention approach can be used for long-lasting obesity prevention, owing to the biological and behavioral plasticity during that developmental period [15,17].

Identification of risk factors for obesity is necessary to establish a basis for preventive action and develop effective interventions. In both adults and children, behavioral factors are well-known modifiable risk factors for obesity [18,19]. Obesity in early childhood involves both genetic and environmental factors [19,20]. Considering the significance of early-life factors in childhood obesity [10,19] and the rapid transitions children experience, a developmental approach for childhood obesity is needed. There is evidence to support the fact that developmental factors contribute significantly to the risk of childhood obesity, with larger size at birth, formula feeding, rapid early growth, less healthy dietary behavior, low physical activity, and high sedentary behavior being identified as developmental risk factors [10]. Thus, it is important to identify the risk factors for obesity in early childhood through a developmental approach that encompasses early-life factors.

Childhood obesity is a complex phenomenon, with a number of mutually influencing factors [21,22,23]. An attempt was made to apply the ecological systems theory to understand these complex risk factors systematically and comprehensively [24,25,26]. According to Davison and Birch’s ecological model, which emphasizes the context of development, childhood overweight is a result of the child’s characteristics and risk factors; parenting style and family characteristics; and community, demographic, and societal characteristics [25]. Child risk factors include dietary intake, physical activity, and sedentary behavior, and their influence on obesity is modulated by the child’s characteristics, including age, sex, and vulnerability to weight gain [25,27]. In addition, child risk factors are influenced by parenting styles and family characteristics such as child feeding practice, types of foods available at home, parental weight status, parental encouragement regarding the child’s activities, parental monitoring of the child’s TV viewing, and the parents’ own behavioral patterns, as well as demographic and societal characteristics, such as socioeconomic status [25,28,29]. A child’s behavioral patterns can become risk factors in the context of their own characteristics, as well as those of the parental and family environment, putting them at risk of obesity. Thus, in order to identify the risk factors for childhood obesity, it is necessary to consider these factors together.

Therefore, in this study, risk factors for obesity in five-year-old children were identified by including their behavioral factors recorded at ages three and five, as well as their characteristics in terms of developmental status and family. Through this, a deeper understanding of the relative contribution of risk factors for early childhood obesity may be gained. Further, by clarifying target behaviors and high-risk groups, the basis for early childhood obesity prevention strategies may be established.

## 2. Materials and Methods

### 2.1. Sample and Data Source

This study was conducted using data from the Korean National Health Insurance Service (NHIS) database. The NHIS is the single insurer in South Korea, covering the entire nation (52,922,000 persons as of September 2021) [30]. Data collected in the process of managing qualifications for insurance premium collection, payment of claim data for medical services, and implementation of health screening services for adults and infants are built into the National Health Insurance Database (NHID). The NHID includes socio-demographic information in the eligibility database; disease and prescription information in the medical services database; and health-related information through physical examination, laboratory tests, and questionnaires in the health screening database [31,32]. The details of the utilization and validity of the NHID are described elsewhere [31,32]. For research purposes, the NHIS provides a customized database for specific populations and related variables in accordance with relevant regulations and procedures. The NHIS-customized database can be accessed and analyzed only within the analysis center provided by NHIS, and online access is not permittable. In the analysis center, only registered researchers can perform analysis on an assigned computer with the analysis software, and results are exported only after approval of the NHIS. For strict confidentiality, the NHID data were anonymized and personal de-identified join keys were assigned to make the linkage between databases and tracking possible. In this study, a series of infant and children health screening databases were connected alongside their mothers’ health screening and eligibility database, which was identified by the mother–child identical health insurance number as well as the same delivery year of the mother and birth year of the child. 

The national health screening program for infants and children in South Korea has been established since November 2007 [33]. The Ministry of Health and Welfare provides a notice of standards for health examination and program information every year; the NHIS sends out health check-up questionnaire sheets and notices by mail at each check-up period. The hospital or health institution designated as an infant and children health screening institution then implements a health check-up and enters the result data [34]. Parents/primary caregivers fill out a questionnaire (which takes about 20 to 30 min and is hence recommended to be completed at home in advance) by referencing the child’s daily life or observing their performance according to instructions, provide information to the doctor during the check-up process, and participate in health counseling [35]. Consultation and developmental evaluation must be performed by a doctor. Health check-ups are scheduled for seven rounds for infants and children from 4 to 71 months of age (1st to 7th: 4–6, 9–12, 18–24, 30–36, 42–48, 54–60, and 66–71 months of age), consisting of anthropometric measurements, physical examination, screening for visual acuity, developmental screening, and general health questionnaires with anticipatory guidance [33,34,36].

Anthropometric measurements (height, weight, BMI, and head circumference) were obtained from physical measurements by a trained personnel of the health screening institution according to the guidelines at each screening: height was measured through an upright standing position (for children over two years old) using a machine with an accuracy of up to 0.1 cm; weight was measured using a machine capable of checking up to 100 g in simple clothes; BMI was calculated as weight (in kilograms) divided by the square of height (in meters); and the sex- and age-specific percentiles for each measurement were calculated using a calculation program according to the 2007 Korean National Growth Charts for children and adolescents [33,34,36,37].

Developmental screening was administered using the Korean Developmental Screening Test for Infants and children (K-DST) tool, which was developed for children from 4 to 71 months of age by the Korean Society of Pediatric Rehabilitation and Developmental Medicine and the Korean Academy of Child and Adolescent Psychiatry in 2014 [38]. Its high validity was confirmed [39]. The K-DST is composed of six domains (gross motor, fine motor, cognition, language, sociality, and self-care). Each domain has eight questions, and each question is scored on a scale of 0–3. The results were interpreted at four different levels: scores above 1 standard deviation (SD) as “advanced development”; between −1 SD and 1 SD as “appropriate for age”; between −2 SD and −1 SD as “follow-up needed (need for follow-up)”; and below −2 SD as “further testing needed (recommendation for further evaluation)” [38,39]. In this study, the parents/primary caregivers recorded the questionnaires and the doctor interpreted and consulted the results.

Parents/primary caregivers also reported their child’s birth weight (checking up to 0.1 kg) and general health information that was asked through a structured questionnaire with several subcategories, one of which was related to nutrition [34,35]. The subcategory of nutrition collected data on dietary and physical behaviors of a child at each screening [34,36]. Since the health screening database provided a series of health screening data for each child, the behaviors at the age of three and five of the same child could be identified and used for study analysis.

This study aimed to determine the influence of behavioral factors of three-year-olds and five-year-olds, as well as child and family characteristics, on the risk of obesity at the age of five. For this purpose, we identified five-year-old children who underwent the sixth screening to confirm obesity, alongside those for whom risk factors could be determined at the age of three through results of the fourth screening test, and family risk factors through mother–child linkage. We initially identified 26,047 children from the 2017 National Health Screening Program who underwent the sixth screening (at age five). The inclusion criteria were as follows: confirmed BMI; availability of an identical mother–child health insurance number, and mother’s delivery and child’s birth being in the same year; and availability of mother’s BMI and income levels. Furthermore, children’s fourth (at age three) health check-up records and birth data from the NHIS database were included.

### 2.2. Variables

The following child characteristics were considered in the analysis: sex, birth weight, and developmental screening at the age of three and five. Birth weight was classified as <2.5 kg, 2.5–4 kg, and ≥4 kg. The results of developmental screening at three and five years of age were classified as “pass” and “caution/refer” according to the decision of the doctor who conducted the Korean Developmental Screening Test [34,36,38].

The following family characteristics were included in the analysis: mother’s BMI, feeding practice, and income level. Mother’s BMI was classified as <25 kg/m^2^ and ≥25 kg/m^2^. Feeding practice was categorized into exclusive breastfeeding, mixed feeding, and formula feeding. Income level was ranked into 21 categories (one category of medical aid beneficiaries and 20 categories of health insurance subscribers) according to income-based insurance premiums, and then divided into three groups for analysis: medical aid and 1 to 6 ranks as “low”; 7 to 13 ranks as “middle”; and 14 to 20 ranks as “high”.

For dietary behavior and physical activity at age three, we selected the nutrition questionnaire from the fourth child health screening. The following items were included in the analysis: child’s appetite (good/average/bad), number of meals per day (one/two/three/four or more), number of snacks per day (one/two/three or more), number of meals with family per week (two days or fewer/three to four days/five days or more), intake of fresh milk per day (none/<200 mL/200–500 mL/500–1000 mL/≥1000 mL), heavy intake of sweet food (yes/no), restricted from eating certain foods owing to allergy concerns (yes/no), and engagement in sweat-inducing physical activities for one hour or more per day (yes/no).

Next, for dietary behavior and physical activity in children aged five at the time of the study, we also included the nutrition questionnaire from the sixth child health screening. The following items were included in the analysis: comparison of eating speed with family (faster/similar/slower), regular meals and snacks (yes/no), quantity of one meal compared to peers (smaller/similar/bigger), eating only what one wants (yes/no), heavy intake of oily and/or salty food (yes/no), preferring other drinks over water (yes/no), watching TV or any other monitor for more than two hours per day (yes/no), and engagement in sweat-inducing physical activities for one hour or more per day (yes/no). 

According to the 2007 Korean National Growth Charts for children and adolescents [33,37], the cutoff for obesity in five-year-old children is a BMI within or above the sex- and age-specific 95th percentile; the non-obesity group includes those with a BMI below the 95th percentile. 

### 2.3. Data Analysis

Statistical analysis was conducted using SAS Enterprise Guide version 7.1 (SAS Institute Inc., Cary, NC, USA). Descriptive statistics, including frequency, percentage, mean, and standard deviation, were calculated for the main variables. To determine the difference between the obesity and non-obesity groups according to the main variables, a chi-square test was performed. The level of significance was set at *p* < 0.05. A hierarchical multiple logistic regression analysis was conducted to examine the relationship between the main variables and obesity at age five using odds ratios (ORs) and 95% confidence intervals (CIs). Variables were then entered into the models using hierarchical entry. Model 1 consisted of child characteristics. Model 2 consisted of child and family characteristics. In Model 3, dietary behavior and physical activity at age three were added to Model 2. Finally, in Model 4, current dietary behavior and physical activity at age five were added to Model 3. The likelihood ratio test for the previous model was analyzed. The R-square was analyzed to confirm the ratio of variance of the dependent variable explained by the independent variables.

### 2.4. Ethical Considerations 

This study was reviewed by the institutional review board of Chodang University (CIRB-2021-07-01) and the NHIS review board (NHIS-2021-1-644), and the informed consent requirement was waived because of secondary analysis of existing anonymized data.

## 3. Results

### 3.1. Subjects’ Characteristics

The subjects’ characteristics are described in Table 1. Among all subjects (*n* = 26,047), 51.1% were male and the mean birth weight was 3.18 kg (SD = 0.46 kg). The proportions of subjects with low birth weight (<2.5 kg) and macrosomia (≥4 kg) were 5.3% and 3.5%, respectively. In the developmental screening at ages three and five, the “caution/refer” remark was made for 13.0% and 16.9% of subjects, respectively. Among mothers, 15.3% were obese, and exclusive breastfeeding was practiced by 40.3%. Regarding financial condition, 19.2% of the children were from low-income households. 

In the distribution of dietary behavior at age three, about half (47.8%) had good appetite. Most subjects (89.2%) had three meals per day. More than half (69.1%) ate snacks twice per day, and 73.1% had meals with family five or more days per week. About half (46.8%) had heavy intake of sweet food and only 14.1% were restricted from eating certain foods because of allergy concerns. Finally, 73.9% engaged in sweat-inducing physical exercise for one hour or more per day.

The indicators of dietary behavior at age five were as follows: about half of the subjects (47%) ate at a similar speed as their family members, most of them (90%) ate meals and snacks regularly, 42.9% ate only what they wanted, 15.7% ate a lot of oily and salty food, and 15.1% preferred other drinks over water. Further, 27.8% of the subjects watched TV or other monitors for more than two hours per day. On the contrary, 75.3% engaged in sweat-inducing physical exercise for one hour or more per day.

### 3.2. Differences between Obesity and Non-Obesity Groups by Main Variables

Table 2 shows the differences between the obesity and non-obesity groups according to the main variables. Compared with the non-obesity group, large birth weight (*p* < 0.001) and the “caution/refer” remark during developmental screening at three years (*p* < 0.001) and five years (*p* < 0.001) were more frequently observed in the obesity group. Obesity among mothers was more common in the obesity group than in the non-obesity group (*p* < 0.001). There were significant differences in terms of breastfeeding (*p* = 0.040) and income level (*p* = 0.001) between the two groups. Exclusive breastfeeding and high level of income were more frequently observed in the non-obesity group.

Among the variables related to dietary behaviors at age three, there were significant differences between the two groups according to appetite (*p* < 0.001), number of weekly meals with family (*p* = 0.029), daily milk consumption (*p* < 0.001), heavy intake of sweet food (*p* < 0.001), and physical exercise for more than one hour (*p* = 0.002).

In the variables related to current dietary behaviors at age five, there were significant differences between the two groups with regard to eating speed (*p* < 0.001), regularity of eating meals and snacks (*p* = 0.002), quantity of a single meal (*p* < 0.001), heavy intake of oily and salty food (*p* < 0.001), and preference for other drinks over water (*p* = 0.007). Moreover, compared to the non-obesity group, the obesity group watched TV or other monitors for more than two hours (*p* < 0.001) and did not engage in physical exercise for more than one hour (*p* = 0.014).

### 3.3. Hierarchical Multiple Logistic Regression Analysis: Age Five

Table 3 shows the results of hierarchical multiple logistic regression analysis of the factors associated with the prevalence of obesity at age five. Four models were fitted.

In Model 1, which included only child characteristics, female sex (OR = 1.21, *p* = 0.001), birth weight of under 2.5 kg (OR = 0.58, *p* < 0.001), birth weight of over 4 kg (OR = 2.02, *p* < 0.001), and the “caution/refer” remark during developmental screening at age three (OR = 1.25, *p* = 0.003) and five (OR = 4.15, *p* < 0.001) had a significant association with the prevalence of obesity at age five. In Model 2, in which family characteristics were added to Model 1, maternal obesity (OR = 2.44, *p* < 0.001) and a middle-level income as compared to high-level income (OR = 1.20, *p* = 0.006) had a significant association with the prevalence of obesity at age five. In Model 3, dietary behaviors and physical activity at age three were added to Model 2. It was found that good appetite (OR = 2.72, *p* < 0.001), bad appetite (OR = 0.31, *p* < 0.001), not consuming any fresh milk (OR = 0.73, *p* = 0.015), milk consumption of 500–1000 mL (OR = 1.65, *p* < 0.001), and heavy intake sweet food (OR = 1.42, *p* < 0.001) were associated with obesity at age five. 

Finally, Model 4 included all variables (child characteristics, family characteristics, dietary behavior at age three, and dietary behavior at age five). It showed that female sex (OR = 1.46, *p* < 0.001), birth weight of less than 2.5 kg (OR = 0.60, *p* = 0.002), birth weight of more than 4 kg (OR = 1.45, *p* = 0.005), the “caution/refer” remark during developmental screening at ages three (OR = 1.26, *p* = 0.005) and five (OR = 4.09, *p* < 0.001), obesity in mothers (OR = 2.13, *p* < 0.001), and a middle-level income (OR = 1.16, *p* = 0.038) were significantly associated with obesity in five-year-old children. However, the ORs of all variables except sex were lower than those in Model 3. In the variables related to dietary behaviors at age three, good appetite (OR = 1.54, *p* < 0.001), milk consumption of 500–1000 mL (OR = 1.50, *p* = 0.007), and heavy intake of sweet food (OR = 1.25, *p* = 0.001) were still significant risk factors for obesity in five-year-old children. On the contrary, having meals with family less frequently than two days per week (OR = 0.76, *p* = 0.032), restrictions on certain foods because of allergies (OR = 0.82, *p* = 0.034), and not engaging in physical exercise for more than one hour (OR = 0.85, *p* = 0.032) significantly lowered the risk of obesity. Lastly, in the variables related to current dietary behavior at age five, speedy eating (OR = 2.60, *p* < 0.001), slow eating (OR = 0.42, *p* < 0.001), irregular meals and snack time (OR = 1.47, *p* < 0.001), smaller single-meal quantities (OR = 0.20, *p* < 0.001), larger single-meal quantities (OR = 4.53, *p* < 0.001), heavy intake of oily and salty food (OR = 1.82, *p* < 0.001), and not performing physical exercise for more than one hour (OR = 1.38, *p* < 0.001) were significantly associated with obesity in five-year-old children.

The difference in likelihood ratio was significant (*p* < 0.001), and the variables added in the model at each stage were confirmed to be significant. Finally, Nagelkerke’s adjusted R-square of Model 4 was 27.2%.

## 4. Discussion

To identify the risk factors for obesity in early childhood, we investigated behavioral factors at ages three and five, child characteristics, and family characteristics while considering developmental aspects. Among these risk factor categories, behavioral factors at age five had the strongest impact on obesity. Further, behavioral factors could have an attenuating effect on the risk of obesity from child and family characteristics. In addition, dietary behaviors, such as food intake and eating habits at ages three and five, and physical activity were identified as risk factors for obesity in five-year-old children. Female sex, large birth weight, maternal obesity, and referral conditions at developmental screening were also risk factors for obesity at age five.

Through hierarchical regression analysis, variable groups of family characteristics (Model 2), behavioral factors at age three (Model 3), and behavioral factors at age five (Model 4) were sequentially added to the child characteristics (Model 1) while proceeding from Model 1 to Model 4. Comparing the increase in the explanatory power of each model according to the model proceeding, the influence of behavioral factors at age five was the largest, followed by the influence of behavioral factors at age three and family characteristics. This indicates the significance of modifiable behavioral factors with regard to obesity, providing evidence that a behavior change approach will be effective in the prevention of obesity in early childhood [15]. 

Looking at each life period, first, large birth weight (included in Model 1) and maternal obesity (added in Model 2) were early-life risk factors for obesity in five-year-old children. Large birth weight and maternal obesity before pregnancy have been suggested as prenatal origins of childhood obesity [40,41,42], and increased body weight at birth also correlates with maternal obesity before pregnancy [43]. Consistent research results affirm that parental obesity, especially maternal obesity, is a risk factor for obesity in children [26,44,45,46,47]. Maternal obesity causes childhood obesity through genetic and environmental influences [48,49,50]. When a child has a genetic tendency toward obesity and the mother creates an obesity-inducing environment via the served food and fostered eating conditions, the expression of such genetic vulnerability is determined [51,52]. Second, as behavioral factors at age three (Model 3), a good appetite and the intake of a large quantity of food in three-year-olds increased the risk of obesity, which was significant even after adding behavioral factors at age five (Model 4), and the OR decreased slightly. In the opposite direction, a low appetite, poor food intake, and meal or food restriction in three-year-olds were significantly associated with a low risk of obesity even after adding behavioral factors in five-year-olds. This indicates that the eating behavior and feeding environment of three-year-olds affects obesity at age five. Third, as behavioral factors at age five (Model 4), the quantity of food intake, dietary patterns, food preferences, and physical activity were related to obesity in five-year-olds, and Model 4 showed an explanatory power of 27% as well as the largest increase compared to previous models, indicating the importance of behavioral factors at age five.

Meanwhile, as behavioral factors were gradually added, it was confirmed that the OR values of birth weight of 4 kg or more, maternal BMI of 25 or more, and middle-level income decreased gradually. This can imply that the effect of large birth weight, maternal obesity, and income level on the child’s obesity risk could be weakened through behavioral factors. It can also be inferred that the obesity risk from child and family characteristics may be attenuated by the change in behavioral factors at ages three and five. In other words, the potential risk of obesity because of child and environmental characteristics can be reduced through behavioral factors, confirming once again the importance of fostering healthy behaviors in children. 

Among behavioral factors, dietary behaviors such as quantity of food intake and eating habits were related to obesity. A good appetite in three-year-olds and a large quantity of food intake compared to peers in five-year-olds increased the risk of obesity by 1.54 times and 4.53 times, respectively. Dietary patterns related to milk and sweet food intake in three-year-olds, as well as oily/salty food intake, irregularity of meals/snacks, and fast eating speed in five-year-olds were significant in obesity at age five. These results emphasize the risk incurred through improper food intake and eating habits, and are consistent with previous studies that confirmed the risk posed by certain dietary behaviors in childhood [28,53,54,55]. When compared with energy consumption, excess energy intake and caloric overload increase the risk of childhood obesity. High-fat foods or energy-dense foods were also identified as risk factors for obesity, indicating the effect of food preferences on childhood obesity. Birch et al. confirmed that it is possible to change children’s food preferences through repeated exposure to healthy food [56]. In addition, based on the results of this study, it can be confirmed that having regular meals/snacks and eating slowly are important for obesity management. Therefore, education on proper eating habits along with exercising control over the amount of food consumed can be helpful in preventing obesity in early childhood. 

Regarding other behavioral factors, not engaging in sweat-inducing physical activities for at least one hour per day was a significant obesity risk factor among five-year-olds, which confirmed the importance of physical activity and balance between energy intake and expenditure [57,58]. However, sedentary behavior (watching TV or other monitors for at least two hours per day) was not a significant factor in this study. Other studies have reported that sedentary behavior increases the risk of childhood obesity [45,59]. The non-significance of TV watching noted in this study could be attributed to the validity of the questions confirming sedentary behavior and the issues of reliability of self-reported responses. Moving forward, the OR for obesity was low for infrequent meals with family (two or fewer times per week) and food restrictions because of allergy concerns. These factors could have affected the quantity and types of meals provided to the children, restricting energy intake to an extent. Another study suggested that parents’ food preparation time could affect the body weight of children, which could have resulted from the effects of how much time parents invested in taking care of the quantity and quality of food provided to their children and in interacting with them [25]. Conversely, the effect of physical activity in three-year-olds appeared to be contradictory to expectation, which could be the result of parents urging their obese children to engage in physical activity. Parents and children mutually influence each other, and parents’ practices are known to be affected by child characteristics such as body weight [25].

However, children’s behavior can be influenced by both their own and family characteristics. In this study, as the behavior factor among five-year-olds was added, the OR of female sex increased in contrast to the decrease in OR of other characteristics, suggesting the possibility of an interaction between sex and the child’s behavior. Being female can influence a child’s physical activity and sedentary lifestyle, leading to differences in obesity risk [17]. In addition, maternal obesity indicated a high risk of obesity in children. Families, including mothers, can act as a behavioral model (through their own preferences and behaviors) and control children’s behavior (through feeding practices and strategies); thus, children can either be at risk for obesity or engage in healthy behaviors [55,56]. It will be helpful for effective child obesity prevention strategies to include changes in parental modeling and practices as important factors, taking into account the complex influence of the characteristics of children, family, and the environment in fostering healthy behaviors in children.

Meanwhile, in this study, conditions requiring “referral” of developmental screening tests at three and five years increased the risk of obesity in children by 1.26 times and 4.09 times, respectively. This is probably because developmental problems, as a basic condition, can affect obesity risk factors. Since the items on developmental factors in this study were very broad, it is difficult to know exactly which developmental issues lead to increased risk of early childhood obesity. As there may be various detailed causes, further studies to confirm the relationship between specific developmental problems in children and obesity are suggested. However, based on the present findings, we suggest that children who require additional monitoring or referral because of developmental problems are the ones who could be considered as requiring obesity management in the future, and thus more attention must be paid to them when creating obesity risk assessments and designing interventions.

In this study, we confirmed the risk factors for obesity in early childhood, which is a critical period in the development of obesity. This study was significant as it compared the influence of each characteristic through a hierarchical analysis while examining child characteristics, family environment characteristics, and behavioral factors of children at ages three and five, since obesity risk is a complex phenomenon in which characteristics, environment, and behavior influence each other. Considering developmental aspects, birth weight, breastfeeding, and developmental screening test variables were included, and follow-up data of behavioral factors of three- and five-year-olds were examined. When defining obesity in five-year-old children, we applied standardized criteria using the BMI percentile for sex and age according to the 2007 Korean National Growth Charts [37]. A large number of subjects were included as a population-based study using the results of infant health check-ups, a basic screening program connected to National Health Insurance in South Korea [31].

This study has certain limitations. As the analysis was limited to data derived from infants and children health check-ups, there was a limitation in the measurement method and variable selection. As the questions on behavioral factors were relatively few, there was a limitation in confirming specific details such as the frequency and intensity of behaviors. In addition, these were measured based on parents’ self-reports and may not reflect the details other than parental observations; there can also be inaccuracy owing to recall and response bias. A study to verify the relevant risk factors through a valid and reliable measurement or intervention is suggested. Moreover, since the behavioral factors of five-year-olds were derived from cross-sectional data, they do not imply a causal relationship; thus, caution is required in the interpretation of our results.

## 5. Conclusions

By identifying the behavioral factors and other characteristics of early childhood obesity risk, the present results expand the understanding of the relative contribution of these factors to early childhood obesity, confirming the rationale for applying cultivation of healthy behavior as a preventive strategy. In addition, babies with high birth weight, children suspected to have developmental conditions, and children with obese mothers can be considered high-risk groups and must be targeted for active obesity prevention interventions. Considering the developmental approach and importance of the familial component, we suggest that parental education and guidance on the creation of a home environment that supports healthy habits need to be integrated into behavioral promotion programs for early childhood obesity prevention. In addition, since obesity risk factors are interrelated, further research is proposed to clarify the relationships among them by using more rigorous methodologies. In the future, we hope that effective strategies, such as efforts to prevent obesity in early childhood and the use of health check-up opportunities, will be developed as prevention interventions.

## Figures and Tables

**Table 1:** Subjects’ characteristics (*n* = 26,047).

Variables		Categories	*n* (%) or Mean ± SD	
**Child characteristics**				
	Sex	Male	13,303	(51.1)
		Female	12,744	(48.9)
	Birth weight (kg)		3.18±	0.46
	Birth weight classification	<2.5 kg	1388	(5.3)
		2.5–4.0 kg	23,745	(91.2)
		≥4.0 kg	914	(3.5)
	Development screening at 3 years	Pass	22,650	(87.0)
		Caution/Refer	3397	(13.0)
	Development screening at 5 years	Pass	21,638	(83.1)
		Caution/Refer	4409	(16.9)
**Family characteristics**				
	Mother’s BMI	<25 kg/m^2^	22,051	(84.7)
		≥25 kg/m^2^	3996	(15.3)
	Feeding practice	Exclusive breastfeeding	10,498	(40.3)
		Mixed feeding	5575	(21.4)
		Formula feeding	9974	(38.3)
	Income level	High	12,720	(48.8)
		Middle	8331	(32.0)
		Low	4996	(19.2)
**Dietary behavior and physical activity at age three**				
	Child’s appetite	Good	12,440	(47.8)
		Average	12,238	(47.0)
		Bad	1369	(5.3)
	Number of meals per day	One	53	(0.2)
		Two	2481	(9.5)
		Three	23,227	(89.2)
		Four or more	286	(1.1)
	Number of snacks per day	One	1831	(7.0)
		Two	18,004	(69.1)
		Three or more	6212	(23.9)
	Number of meals with family per week	Two days or fewer	2151	(8.3)
		Three to four days	4845	(18.6)
		Five days or more	19,051	(73.1)
	Intake of fresh milk per day	None	1932	(7.4)
		<200 mL	12,377	(47.5)
		200–500 mL	10,848	(41.7)
		500–1000 mL	847	(3.3)
		≥1000 mL	43	(0.2)
	Heavy intake of sweet food	Yes	12,195	(46.8)
		No	13,852	(53.2)
	Restricted from eating certain foods owing to allergy concerns	Yes	3679	(14.1)
		No	22,368	(85.9)
	Engagement in sweat-inducing physical activities for 1 h or more per day	Yes	19,241	(73.9)
		No	6806	(26.1)
**Dietary behavior and physical activity at age five**				
	Comparison of eating speed with family	Faster	907	(3.5)
		Similar	12,253	(47.0)
		Slower	12,887	(49.5)
	Regular meals and snacks	Yes	23,441	(90.0)
		No	2606	(10.0)
	Quantity of one meal compared to peers	Smaller	4974	(19.1)
		Similar	18,981	(72.9)
		Bigger	2092	(8.0)
	Eating only what one wants	Yes	11,169	(42.9)
		No	14,878	(57.1)
	Heavy intake of oily and salty food	Yes	4087	(15.7)
		No	21,960	(84.3)
	Preferring other drinks over water	Yes	3939	(15.1)
		No	22,108	(84.9)
	Watching TV or any other monitor for more than 2 h per day	Yes	7246	(27.8)
		No	18,801	(72.2)
	Engagement in sweat-inducing physical activities for 1 h or more per day	Yes	19,604	(75.3)
		No	6443	(24.7)

**Table 2:** Differences between obesity and non-obesity groups according to the main variables (*n* = 26,047).

Variables		Categories	Obesity (*n* = 1328)		Non-Obesity Group (*n* = 24,719)		t or X^2^	*p*-Value
			*n* (%) or Mean ± SD		*n* (%) or Mean ± SD			
**Child characteristics**							3.10	0.078
	Sex	Male	647	(48.7)	12,656	(51.2)		
		Female	681	(51.3)	12,063	(48.8)	−9.23	<0.001
	Birth weight (kg)		3.29±	0.47	3.17±	0.46	46.78	<0.001
	Birth weight classification	<2.5 kg	48	(3.6)	1340	(5.4)		
		2.5–4.0 kg	1192	(89.8)	22,553	(91.2)		
		≥4.0 kg	88	(6.6)	826	(3.3)	78.32	<0.001
	Development screening at 3 years	Pass	1049	(79.0)	21,601	(87.4)		
		Caution/Refer	279	(21.0)	3118	(12.6)	720.00	<0.001
	Development screening at 5 years	Pass	746	(56.2)	20,892	(84.5)		
		Caution/Refer	582	(43.8)	3827	(15.5)		
**Family characteristics**							245.02	<0.001
	Mother’s BMI	<25 kg/m^2^	924	(69.6)	21,127	(85.5)		
		≥25 kg/m^2^	404	(30.4)	3592	(14.5)	6.43	0.040
	Feeding practice	Exclusive breastfeeding	496	(37.4)	10,002	(40.5)		
		Mixed feeding	283	(21.3)	5292	(21.4)		
		Formula feeding	549	(41.3)	9425	(38.1)	13.42	0.001
	Income level	High	588	(44.3)	12,132	(49.1)		
		Middle	479	(36.1)	7852	(31.8)		
		Low	261	(19.7)	4735	(19.2)		
**Dietary behavior and physical activity at age three**								300.64
	Child’s appetite	Good	937	(70.6)	11,503	(46.5)		
		Average	375	(28.2)	11,863	(48.0)		
		Bad	16	(1.2)	1353	(5.5)	6.26	0.100
	Number of meals per day	One	3	(0.2)	50	(0.2)		
		Two	115	(8.7)	2366	(9.6)		
		Three	1187	(89.4)	22,040	(89.2)		
		Four or more	23	(1.7)	263	(1.1)	1.31	0.520
	Number of snacks per day	One	83	(6.3)	1748	(7.1)		
		Two	927	(69.8)	17,077	(69.1)		
		Three or more	318	(24.0)	5894	(23.8)	7.10	0.029
	Number of meals with family per week	Two days or fewer	86	(6.5)	2065	(8.4)		
		Three to four days	267	(20.1)	4578	(18.5)		
		Five days or more	975	(73.4)	18,076	(73.1)	31.18	<0.001
	Intake of fresh milk per day	None	76	(5.7)	1856	(7.5)		
		<200 mL	581	(43.8)	11,796	(47.7)		
		200–500 mL	599	(45.1)	10,249	(41.5)		
		500–1000 mL	70	(5.3)	777	(3.1)		
		≥1000 mL	2	(0.2)	41	(0.2)	16.17	<0.001
	Heavy intake of sweet food	Yes	693	(52.2)	11,502	(46.5)		
		No	635	(47.8)	13,217	(53.5)	3.33	0.068
	Restricted from eating certain foods owing to allergy concerns	Yes	165	(12.4)	3514	(14.2)		
		No	1163	(87.6)	21,205	(85.8)	9.47	0.002
	Engagement in sweat-inducing physical activities for 1 h or more per day	Yes	1029	(77.5)	18,212	(73.7)		
		No	299	(22.5)	6507	(26.3)		
**Dietary behavior and physical activity at age five**								1108.95
	Comparison of eating speed with family	Faster	235	(17.7)	672	(2.7)		
		Similar	818	(61.6)	11,435	(46.3)		
		Slower	275	(20.7)	12,612	(51.0)	9.67	0.002
	Regular meals and snacks	Yes	1162	(87.5)	22,279	(90.1)		
		No	166	(12.5)	2440	(9.9)	1749.29	<0.001
	Quantity of one meal compared to peers	Smaller	37	(2.8)	4937	(20.0)		
		Similar	792	(59.6)	18,189	(73.6)		
		Bigger	499	(37.6)	1593	(6.4)	0.77	0.379
	Eating only what one wants	Yes	554	(41.7)	10,615	(42.9)		
		No	774	(58.3)	14,104	(57.1)	149.03	<0.001
	Heavy intake of oily and salty food	Yes	366	(27.6)	3721	(15.1)		
		No	962	(72.4)	20,998	(85.0)	7.22	0.007
	Preferring other drinks over water	Yes	235	(17.7)	3704	(15.0)		
		No	1093	(82.3)	21,015	(85.0)	36.09	<0.001
	Watching TV or any other monitor for more than 2 h per day	Yes	465	(35.0)	6781	(27.4)		
		No	863	(65.0)	17,938	(72.6)	6.00	0.014
	Engagement in sweat-inducing physical activities for 1 h or more per day	Yes	962	(72.4)	18,642	(75.4)		
		No	366	(27.6)	6077	(24.6)	3.10	0.078

**Table 3:** Hierarchical multiple logistic regression analysis of obesity among subjects at age five (*n* = 26,047).

Variables		Categories	Model 1		Model 2		Model 3		Model 4	
			OR (95% CI)	*p*-Value	OR (95% CI)	*p*-Value	OR (95% CI)	*p*-Value	OR (95% CI)	*p*-Value
**Child characteristics**										
	Sex	Male	Ref.		Ref.		Ref.		Ref.	
		Female	1.21 (1.08–1.35)	0.001	1.20 (1.07–1.34)	0.002	1.21 (1.08–1.36)	0.001	1.46 (1.29–1.65)	<0.001
	Birth weight classification	<2.5 kg	0.58 (0.43–0.78)	<0.001	0.54 (0.40–0.73)	<0.001	0.56 (0.41–0.76)	<0.001	0.60 (0.43–0.82)	0.002
		2.5–4.0 kg	Ref.		Ref.		Ref.		Ref.	
		≥4.0 kg	2.02 (1.60–2.56)	<0.001	1.74 (1.37–2.20)	<0.001	1.59 (1.25–2.02)	<0.001	1.45 (1.12–1.88)	0.005
	Development screening at 3 years	Pass	Ref.		Ref.		Ref.		Ref.	
		Caution/Refer	1.25 (1.08–1.44)	0.003	1.21 (1.05–1.41)	0.01	1.34 (1.15–1.55)	<0.001	1.26 (1.07–1.48)	0.005
	Development screening at 5 years	Pass	Ref.		Ref.		Ref.		Ref.	
		Caution/Refer	4.15 (3.69–4.67)	<0.001	4.12 (3.66–4.65)	<0.001	4.24 (3.76–4.79)	<0.001	4.09 (3.59–4.66)	<0.001
**Family characteristics**										
	Mother’s BMI	<25 kg/m^2^			Ref.		Ref.		Ref.	
		≥25 kg/m^2^			2.44 (2.15–2.77)	<0.001	2.35 (2.06–2.67)	<0.001	2.13 (1.85–2.45)	<0.001
	Feeding practice	Exclusive breastfeeding			Ref.		Ref.		Ref.	
		Mixed feeding			1.08 (0.93–1.26)	0.323	1.08 (0.93–1.26)	0.325	1.08 (0.92–1.28)	0.349
		Formula feeding			1.10 (0.97–1.25)	0.14	1.11 (0.98–1.27)	0.109	1.14 (0.99–1.31)	0.061
	Income level	High			Ref.		Ref.		Ref.	
		Middle			1.20 (1.05–1.36)	0.006	1.18 (1.04–1.35)	0.010	1.16 (1.01–1.33)	0.038
		Low			1.03 (0.88–1.20)	0.694	1.00 (0.86–1.17)	0.976	0.97 (0.82–1.14)	0.692
**Dietary behavior and physical activity at age three**										
	Child’s appetite	Good					2.72 (2.39–3.09)	<0.001	1.54 (1.34–1.77)	<0.001
		Average					Ref.		Ref.	
		Bad					0.31 (0.19–0.51)	<0.001	0.55 (0.32–0.92)	0.024
	Number of meals per day	One					1.39 (0.41–4.70)	0.594	0.95 (0.25–3.67)	0.945
		Two					1.10 (0.89–1.36)	0.360	1.00 (0.80–1.25)	0.983
		Three					Ref.		Ref.	
		Four or more					1.01 (0.64–1.60)	0.956	0.80 (0.49–1.32)	0.384
	Number of snacks per day	One					Ref.		Ref.	
		Two					0.98 (0.77–1.25)	0.878	1.00 (0.77–1.30)	0.989
		Three or more					0.85 (0.65–1.10)	0.212	0.85 (0.64–1.12)	0.244
	Number of meals with family per week	Two days or fewer					0.81 (0.64–1.03)	0.082	0.76 (0.60–0.98)	0.032
		Three to four days					1.13 (0.97–1.30)	0.109	1.09 (0.93–1.27)	0.283
		Five days or more					Ref.		Ref.	
	Intake of fresh milk per day	None					0.73 (0.57–0.94)	0.015	0.64 (0.49–0.83)	0.001
		<200 mL					0.89 (0.78–1.00)	0.052	0.89 (0.78–1.01)	0.08
		200~500 mL					Ref.		Ref.	
		500~1000 mL					1.65 (1.26–2.17)	<0.001	1.50 (1.12–2.02)	0.007
		≥1000 mL					0.70 (0.16–3.00)	0.632	0.48 (0.09–2.63)	0.398
	Heavy intake of sweet food	Yes					1.42 (1.26–1.59)	<0.001	1.25 (1.10–1.42)	0.001
		No					Ref.		Ref.	
	Restricted from eating certain foods owing to allergy concerns	Yes					0.86 (0.72–1.02)	0.077	0.82 (0.68–0.99)	0.034
		No					Ref.		Ref.	
	Engagement in sweat-inducing physical activities for 1 h or more per day	Yes					0.90 (0.79–1.04)	0.146	0.85 (0.73–0.99)	0.032
		No								
**Dietary behavior and physical activity at age five**										
	Comparison of eating speed with family	Faster							2.60 (2.15–3.16)	<0.001
		Similar							Ref.	
		Slower							0.42 (0.36–0.49)	<0.001
	Regular meals and snacks	Yes							Ref.	
		No							1.47 (1.20–1.79)	<0.001
	Quantity of one meal compared to peers	Smaller							0.20 (0.14–0.29)	<0.001
		Similar							Ref.	
		Bigger							4.53 (3.93–5.21)	<0.001
	Eating only what one wants	Yes							1.08 (0.94–1.23)	0.275
		No							Ref.	
	Heavy intake of oily and salty food	Yes							1.82 (1.57–2.13)	<0.001
		No							Ref.	
	Preferring other drinks over water	Yes							1.17 (0.99–1.38)	0.064
		No							Ref.	
	Watching TV or any other monitor for more than 2 h per day	Yes							1.11 (0.97–1.27)	0.14
		No							Ref.	
	Engagement in sweat-inducing physical activities for 1 h or more per day	Yes							Ref.	
		No							1.38 (1.20–1.59)	<0.001
Log-likelihood			−4934.13		−4839.45		−4640.13		−4017.65	
X^2^ (*p*-value)			623.71	<0.001	813.08	<0.001	1211.72	<0.001	2456.67	<0.001
AIC			9880.262		9700.895		9334.252		8109.307	
Nagelkerke’s adjusted R-Square			0.071		0.093		0.137		0.272	

OR, odds ratio; Ref., reference; AIC, Akaike’s information criterion.

## Data Availability

All available data generated or analyzed during this study are included in this article. The original research data are not available due to the strict confidentiality guidelines and regulations by the National Health Insurance Service. Access to research data is permitted only at the designated analysis center by the designated researcher and data sharing is strictly prohibited.
